# A Practical Framework for the Integration of Structural Data Into Perimetric Examinations

**DOI:** 10.1167/tvst.13.6.19

**Published:** 2024-06-25

**Authors:** Josephine C. Evans, Giovanni Ometto, David P. Crabb, Giovanni Montesano

**Affiliations:** 1 City, University of London, Optometry and Visual Sciences, London, UK; 2 NIHR Biomedical Research Centre, Moorfields Eye Hospital NHS Foundation Trust and UCL Institute of Ophthalmology, London, UK; 3 London North West Healthcare NHS Trust, Harrow, London, UK

**Keywords:** visual field, perimetry, OCT, structure, application

## Abstract

**Purpose:**

We sought to develop and evaluate a practical framework that supports structurally enhanced perimetric examinations.

**Methods:**

Two perimetric strategies were compared: standard Zippy Estimation through Sequential Testing (ZEST) procedure, a traditional visual field test with population-based prior distributions, and structural-ZEST (S-ZEST), enhanced with individual optical coherence tomography data to determine the starting parameters. The integration and collection of data was facilitated by a bespoke application developed in Shiny R (R Studio). The test was implemented using the Open Perimetry Interface on the Compass perimeter (CentreVue-iCare, Italy). The strategies were evaluated via simulations and on 10 visually healthy participants. The usability of the application was assessed in a simulated environment with 10 test users.

**Results:**

In simulations, the S-ZEST improved test speed in patients with glaucoma. In the practical implementation, there was a statistically significant decrease in the testing time (approximately 26%) and in the number of presentations per test with S-ZEST (*P* < 0.001). The structure–function relationship was similar between the two strategies. The time taken for users to complete the sequence of actions on the application was 52.9 ± 11.5 seconds (mean ± standard deviation).

**Conclusions:**

Structurally enhanced perimetric examination can significantly improve test time in healthy subjects and can be delivered through a user-friendly interface. Further testing will need to assess feasibility and performance of S-ZEST in patients with glaucoma.

**Translational Relevance:**

We have developed a user-friendly web application based within the Shiny environment for R, which implements an automated extraction of optical coherence tomography data from raw files and performs real-time calculations of structural features to inform the perimetric strategy.

## Introduction

Central visual field (VF) assessment with standard automated perimetry (SAP) is fundamental to glaucoma diagnosis and monitoring.^1^ Nonetheless, SAP is onerous and time consuming for patients^2^ and suffers from considerable test–retest variability, particularly in individuals with decreased VF sensitivity.^3^^–^^6^ Improving the speed and reliability of VF testing is advantageous both clinically and for patient experience. Progress has been made with the development of faster strategies^7^; however, this is often accompanied by a loss of accuracy.^5^^,^^8^

The macula contains almost one-half of the total retinal ganglion cells in healthy eyes and can be affected by glaucoma, even in its early stages.^9^ Despite this, the macula is only sparsely sampled by the most common 24-2 VF testing pattern and subsequently these defects often go undetected.^9^^,^^10^ Other VF testing patterns, such as the 10-2, can provide a more accurate mapping of this region and uncover VF defects that can have a significant impact on patients’ quality of life.^11^ Macular damage can also be assessed with structural imaging. Spatially detailed structural maps can be easily obtained with spectral domain optical coherence tomography (SD-OCT), which is used routinely in glaucoma care.^1^ Typically, in clinics, SD-OCT data and SAP tests are obtained separately and reviewed side by side. However, structural data can be used to inform the execution of SAP in experimental settings, improving both the speed and reliability of the test.^12^^–^^14^

The Compass (CMP, CentreVue-iCare, Vignoza, Italy) is a fundus tracking perimeter that uses live imaging of the retina to record and compensate for eye movements. This methodology improves the spatial accuracy of the test, reducing the reliance on a patient's stable fixation at a central target.^15^ The Compass has been shown to have equivalent diagnostic precision to the Humphrey Field Analyzer (Zeiss, Dublin, CA)^16^ and improved test-retest variability for the measurement of mean sensitivity. Besides its native commercial software, the CMP can be controlled through the Open Perimetry Interface (OPI),^17^^,^^18^ which facilitates the practical implementation and evaluation of novel experimental testing procedures.

The purpose of this study was to develop and evaluate a practical framework using an interactive application that enables structurally enhanced SAP tests. SD-OCT scans were performed on visually healthy volunteers and used to generate prior knowledge for a Bayesian threshold strategy using a 10-2 test pattern. The VF test was implemented using the OPI on the CMP. This structurally enhanced strategy was compared with a traditional Bayesian threshold strategy, informed by population values, for both test–retest variability and test duration.

The workflow and the integration of structural data was facilitated by a bespoke application that could import the data and run the perimetric test. Previous attempts to integrate structural information into VF testing have been limited by the absence of a user-friendly interface that can be applied in a clinical or research setting.^12^^–^^14^ This web-based application, based within Shiny environment for R, offers real-time calculations of structural features to facilitate the integration and delivery of structurally enhanced VF tests. This study examines the usability and implementation of this application.

## Methods

### Bayesian Perimetric Strategies and Simulations

Our perimetric strategies were implementations of the Zippy Estimation through Sequential Testing (ZEST) procedure.^19^ Our procedure was based on the implementation of ZEST provided within the OPI package^17^^,^^18^ for R (R Foundation for statistical computing, Vienna, Austria). Briefly, a prior distribution of possible outcome values between 0 dB and 40 dB is updated using a likelihood function based on the responses from the subject to each presentation (seen or not seen). The likelihood function is the cumulative distribution function (*cdf*) of a Gaussian with a 1-dB standard deviation and asymptotes at 0.03 and 0.97, centered on the intensity value being tested. For each location, the mean of the updated prior distribution (posterior distribution) is used to determine the intensity of the presentation in the next step. The strategy terminates when all locations have reached a termination criterion (standard deviation of the posterior distribution of ≤1.5 dB).

**Figure 1.**
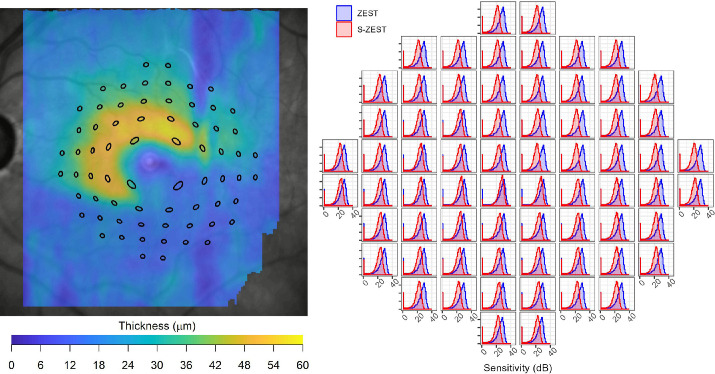
Example of the structure–function model in a glaucoma patient. The graphs on the right are plotted against sensitivity. The peaks of the standard ZEST (*blue*) are centered on the normative values and the S-ZEST priors (*red*) are centered on the prediction of the SF model. For a glaucoma patient, where the GCL is thinner (inferiorly on the fundus image), the corresponding peaks of S-ZEST are shifted toward the lower sensitivities. Test position of the test locations accounts for the displacement of the retinal ganglion cells in the macula.

The prior distributions for the traditional ZEST were generated using a 4:1 mixture of normal and abnormal thresholds, derived from Turpin et al.^20^ The peak of the distribution, identified as the mode, of normal thresholds was centered on normative values for the 10-2 grid for the CMP, which was obtained by linearly interpolating the data collected in a validation study.^16^ The structurally enhanced ZEST (S-ZEST) was identical, but the peak of normal thresholds was shifted to be centered on the value predicted by a point-wise macular structure-function model, similar to one used elsewhere,^12^ using the thickness of the ganglion cell layer (GCL). An example for a patient with glaucoma in the simulation dataset is shown in Figure 1.

The structure–function model was also used to alter the mixing proportion of the normal and abnormal component. The details of the structure–function model and of the generation of the structural prior distributions are reported in the Supplementary Material. The parameters of the model were estimated from test–retest data collected in a cohort of patients with glaucoma during a separate study.^21^ A description of this dataset is provided in the Supplementary Material.

The two strategies were first evaluated via simulations using 10-2 data from 21 visually healthy subjects and 32 glaucoma subjects, collected with the CMP for a previously published study.^12^ Note that these numbers are slightly different from those reported in Montesano et al.^12^ because we were not able to retrieve the presentation history for two tests, required for the original implementation of the model but not for its current version. The original data collection recruited more patients with advanced glaucoma to maximize the likelihood of detecting glaucomatous damage. The sample contained 19 eyes with advanced loss (24-2 mean deviation [MD] of < −12 dB), 8 eyes with moderate loss (24-2 MD of < −6 dB), and 5 eyes with early loss (24-2 MD of ≥ −6 dB). The characteristics are reported in Table 1.

**Table 1.** Characteristics of the Subjects in the Testing Set Used for Simulations, Reported as Average (Standard Deviation)

	Age (Years)	MD 24-2 (dB)	PSD 24-2 (dB)	MS 10-2 (dB)	Visual Acuity (Decimals)
Glaucoma (*N* = 32)	69 (14)	−13.8 (7.46)	9.17 (3.42)	14.64 (6.19)	0.71 (0.26)
Healthy (*N* = 21)	47 (16)	–	–	28.89 (1.49)	0.97 (0.07)

MS, mean sensitivity; PSD, pattern standard deviation.

These simulations tested two variations of ZEST and S-ZEST, namely, with and without spatial enhancement. The spatial correlations were implemented as described in Rubinstein et al.^22^ For ZEST, we connected the nearest neighbors and the strength of the correlation was 0.2. Locations were disconnected across the horizontal midline. For S-ZEST, the correlation was stronger (0.4), but neighboring locations were disconnected if the sensitivity predicted by the structure–function model differed by more than 1 dB.

We simulated both reliable (5% false-positive and false-negative responses) and unreliable observers (20%). Simulations were implemented using the OPI package. Responses were generated using a Gaussian psychometric function whose slope (standard deviation) varied according to sensitivity as described by Henson et al.^23^ and as implemented in the OPI package. Simulations were repeated 500 times for each eye in the dataset.

## Practical Implementation of the Test

### Subject Recruitment

Data from 10 healthy participants were collected at City, University of London. The protocol obtained ethical approval from City's research ethics committee (reference number ETH2021-1823) and adhered to the tenets of the Declaration of Helsinki. Participants had to be older than 18 years of age, provide informed consent, have a spherical prescription between −10 D and + 6 D, astigmatism less than ±2 D, and an intraocular pressure of less than 21 mm Hg. Participants were excluded if they had ocular or systemic conditions that may affect the VF test. One eye was tested per subject, chosen at random if both were eligible. Volunteers were compensated for their participation. (The study was funded by the Higher Education Innovation Fund from City, University of London.)

In the first session, preliminary examination involved ocular and systemic medical history, BCVA (logarithm of the minimum angle of resolution), noncontact tonometry and autorefraction (Topcon, Tokyo, Japan, TRK-1P), and ocular examination (biomicroscope). All subjects had macular and optic nerve head (ONH) scans with a Spectralis SD-OCT (Heidelberg Spectralis, Heidelberg, Germany). This device uses a dual beam scanning laser ophthalmoscopy (SLO) technology to track and compensate for eye movements during the scan. OCT data were collected with the premium glaucoma module, which automatically centers the acquisition of the macular scans on the anatomical fovea and the ONH scans on the center of the Bruch's membrane opening. Macular scans contained 61 B-scans (spanning an 30° × 25° area, 9 averaged acquisitions). The scans were automatically orientated along the fovea–disc axis. The ONH scans contained 25 radial scans of the nerve and 3 concentric circumpapillary scans. The .vol files were generated with a version of the Heyex software (Heidelberg Engineering, Heidelberg, Germany) and enabled for RAW export of the OCT data.

### Perimetric Test

Perimetric data were collected with the CMP fundus perimeter using the OPI. The device uses continuous imaging of the retina (25 frames/second) to help compensate for eye movements during the test. The CMP also captures an image at the beginning of the exam, which enables anatomical landmarks to be used as a reference to target specific areas of the retina during the perimetric test. Because images of the retina are collected during the examination, the CMP is equipped with autofocus and no correction lens is needed.

**Figure 2.**
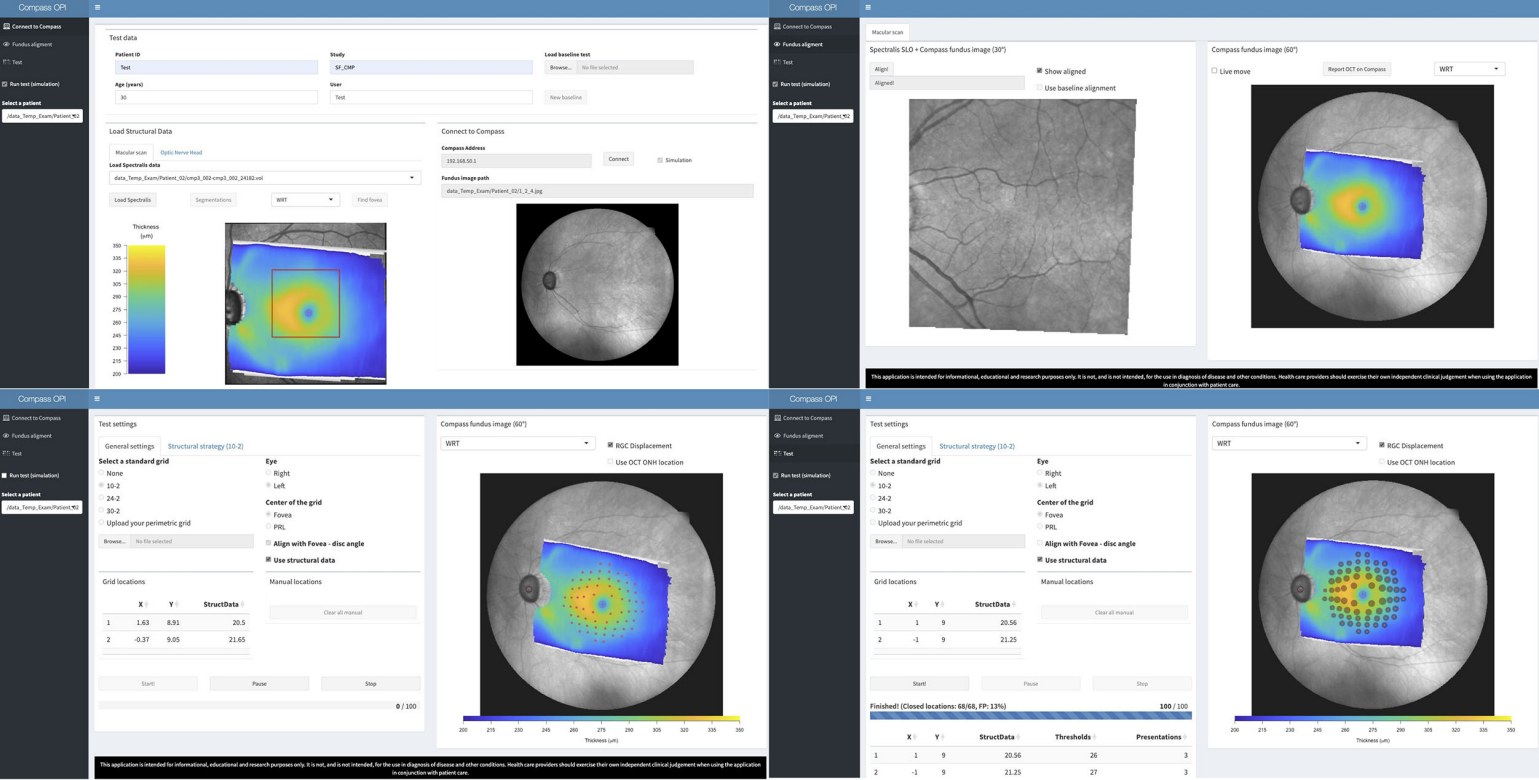
Image of the Shiny R application. A video demonstrating the application can be accessed on https://youtu.be/wOp8zp2BHcs. The application is available on https://github.com/giovmontesano/shinyCMP_OPI.

The collection of VF data was facilitated by a bespoke application developed in Shiny R (R Studio), which is based on the open source environment R. The application connected to the CMP via the OPI and was able to read the RAW OCT files. Both the macular and the ONH scans were imported in the application. The fovea was located automatically on the OCT scan by detecting the point of maximum normalized cross-correlation between the retinal thickness map and a template of the fovea generated as the average of 30 healthy scans, previously collected for a separate study. The search region for the cross-correlation was restricted to a 12.5° × 12.5° region, initially located on the center of SLO, but that could be manually relocated by the user if needed (Fig. 2, top left).

The application then automatically aligned the SLO images from the Spectralis with the fundus infrared reference image acquired by the CMP at the beginning of the test (Fig. 2, top right). This process allowed accurate mapping of the macular thickness on the CMP reference images. The alignment was performed with the package RNiftyReg, an interface for the NiftyReg registration tool, and matching was achieved by cropping the area covering the central 30 × 30 visual degree area from the Compass image. The cropped image was then rescaled (linear interpolation) to match the resolution of the Spectralis SLO. The cropped area from the Compass was initially centered on the center of the CMP fundus image, but the user can manually select a different center to improve the initial alignment. The user then selected an alignment button to calculate an affine transformation.

The fovea was used as the center for the 10-2 grid. The center of the ONH was extracted from the ONH OCT scan. The grid was rotated so that the midline was orientated along the fovea–disc axis (Fig. 2, bottom left). The average thickness of the GCL corresponding with each location was calculated automatically by the application after compensating for ganglion cell displacement using the implementation of the Drasdo model^24^ proposed by Montesano et al.^25^ These thickness values were used to predict the expected sensitivity using the point-wise structure–function model. The predicted sensitivity informed the prior distributions of the S-ZEST, as previously described (Fig. 2, bottom right).

Participants completed four VF tests over two sessions; the test was repeated twice for both strategies, ZEST and S-ZEST, to assess test–retest variability. The order of the four tests was randomized computationally. The sessions were scheduled on separate days and there was a break of at least 10 minutes between tests. The image alignment and calculation of the GCL thickness was performed for the first, baseline test only, regardless of the strategy selected. The application was able to execute follow-up tests, which used the reference image and structural data from the baseline test to assess the same locations in all follow-ups. The application allowed the user to start, pause, and stop the test if needed.

The protocol specified a threshold of 15% false-positive responses, with a chance to repeat the test if unreliable. However, none of the tests had to be excluded or repeated for exceeding this threshold.

### Statistical Analysis

Bland–Altman analysis^26^ was used to assess point-wise test–retest variability. Confidence intervals (CIs) for the 95% limits of repeatability and the significance of the decrease in their amplitude was quantified via bootstrap, where subjects were the sampling unit. Pairwise agreement between individual repetitions of ZEST and S-ZEST are also reported, with the corresponding 95% limits of agreement. Differences in test duration, number of presentations, and the structure–function correlation with local log10(GCL thickness) measurements were tested with linear mixed models. For the structure–function correlation, both random slopes and random intercepts were used. The R^2^ for the structure-function correlation was calculated only using the prediction from the fixed effects. The mean absolute error was used to summarize the results of the simulations. No statistical testing was performed on the simulation results because the sample size of a simulation can be increased arbitrarily to reach any level of significance.

### Usability of the Application

Usability was assessed by 10 participants in a simulation environment for the application. The participants worked in the field of clinical ophthalmic care or vision science, with a range of expertise (three clinicians, three researchers, two ophthalmic technicians, one student of optometry, and one teaching academic). None of the participants had prior experience with the application and they were not involved in its design or conceptualization.

The simulation used data from the 19 glaucoma eyes used to derive the structure–function model (see the Supplementary Material). The OCT, fundus photographs and alignments would therefore be representative of those encountered in a typical cohort of patients. The participants were asked to complete a specific sequence of actions: (1) upload the OCT data and the Compass fundus photograph, (2) Identify the fovea using the semiautomated algorithm, (3) align the OCT thickness maps and the Compass fundus photograph using the semi-automated tool, (4) select the correct test pattern (10-2) and select the alignment of the grid along the fovea-disc axis, (5) calculate the average GCL thickness for all locations using the automated tool, (6) select the option to use S-ZEST for testing, and (7) start the test.

The correct completion of the sequence was recorded by the application and the correct alignment of the OCT maps was verified by the investigator at the end of the sequence. The application also recorded the time taken for the task to be completed (on a MacBook Air M1). For a subset of 5 of 19 patients, randomly chosen for each participant, the user was asked to start, pause, and restart the test in a simulated setting. An artificial delay was added to allow time for the user to perform these actions. These five tests were excluded from the computation of the average procedure time.

## Results

### Simulations

**Figure 3.**
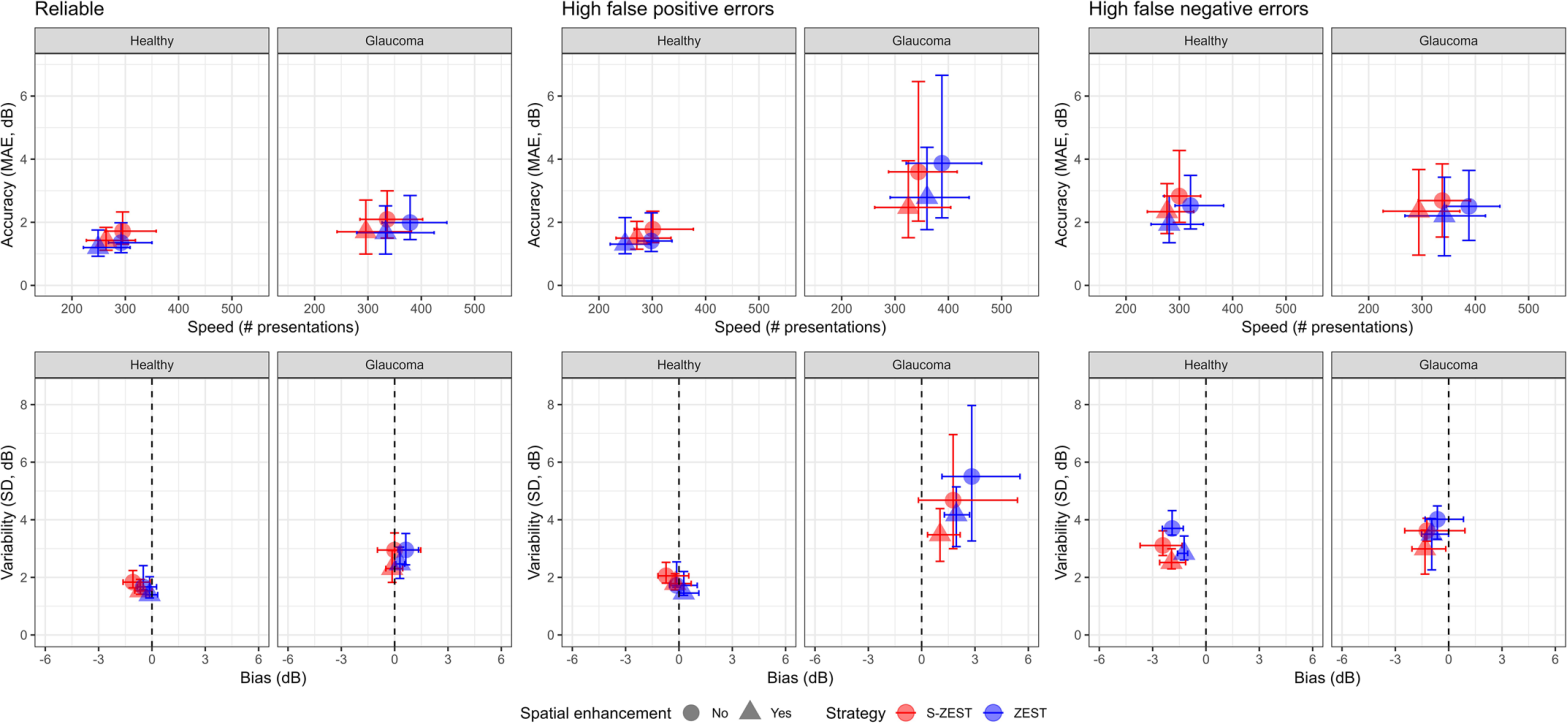
Results of the simulations in healthy observers and patients with glaucoma, in addition to reliable and unreliable observers. MAE, mean absolute error per test. High false-positive and -negative errors = 20%.

The simulations tested the four possible implementations of the strategy: with and without structural information and with and without spatial enhancement, in which neighboring locations were used to help determine the final threshold for a location. The bars in Figure 3 represent the 2.5% and 97.5% quantiles of the results of the simulations (500 simulations, 53 tests). As currently implemented, the S-ZEST provided little to no advantage in accuracy, but it did improve test speed in patients with glaucoma. Spatial enhancement further improved speed in patients with glaucoma, with similar accuracy as ZEST. In healthy subjects, the combination of S-ZEST and spatial enhancement provided an improvement in speed. The accuracy for healthy subjects was slightly reduced with S-ZEST. This was expected because the structure–function model was estimated using data only from patients with glaucoma. As expected, data from healthy individuals demonstrates faster speed and lower error. The numerical values for Figure 3 are provided in Supplementary Material.

### Practical Implementation

Participant demographics and preliminary examination results are summarized in Table 2.

**Table 2.** Participant Information

Participant (SF-CMP_)	Age (Years)	Gender	Eye	Autorefraction	Visual Acuity (LogMAR)	Intraocular Pressure (mm Hg)
1	33	Female	Left	−3.00/−1.00 × 154	0.02	16
2	27	Male	Right	−5.75/−0.75 × 173	−0.1	12
3	25	Female	Right	+0.25/−0.75 × 31	−0.1	13
4	33	Male	Left	−3.25−0.25 × 11	0.02	18
5	26	Female	Right	−0.25 DS	0.02	11
6	27	Male	Left	−0.25/−0.50 × 171	−0.1	14
7	36	Male	Right	+0.25/−1.25 × 3	0.00	19
8	25	Male	Left	+0.75/−0.75 × 8	0.00	15
9	28	Female	Right	−2.25/−0.50 × 43	0.00	15
10	26	Male	Right	−0.75/−0.25 × 7	0.02	16

LogMAR, logarithm of the minimum angle of resolution.

All participants completed two repetitions of the tests performed with ZEST and S-ZEST strategies. Based on the results from the simulations, the participants were tested using ZEST without spatial enhancement and a S-ZEST with spatial enhancement, because these are the combinations that are likely to make the largest difference for patients and have the most translational value (see Discussion).

**Figure 4.**
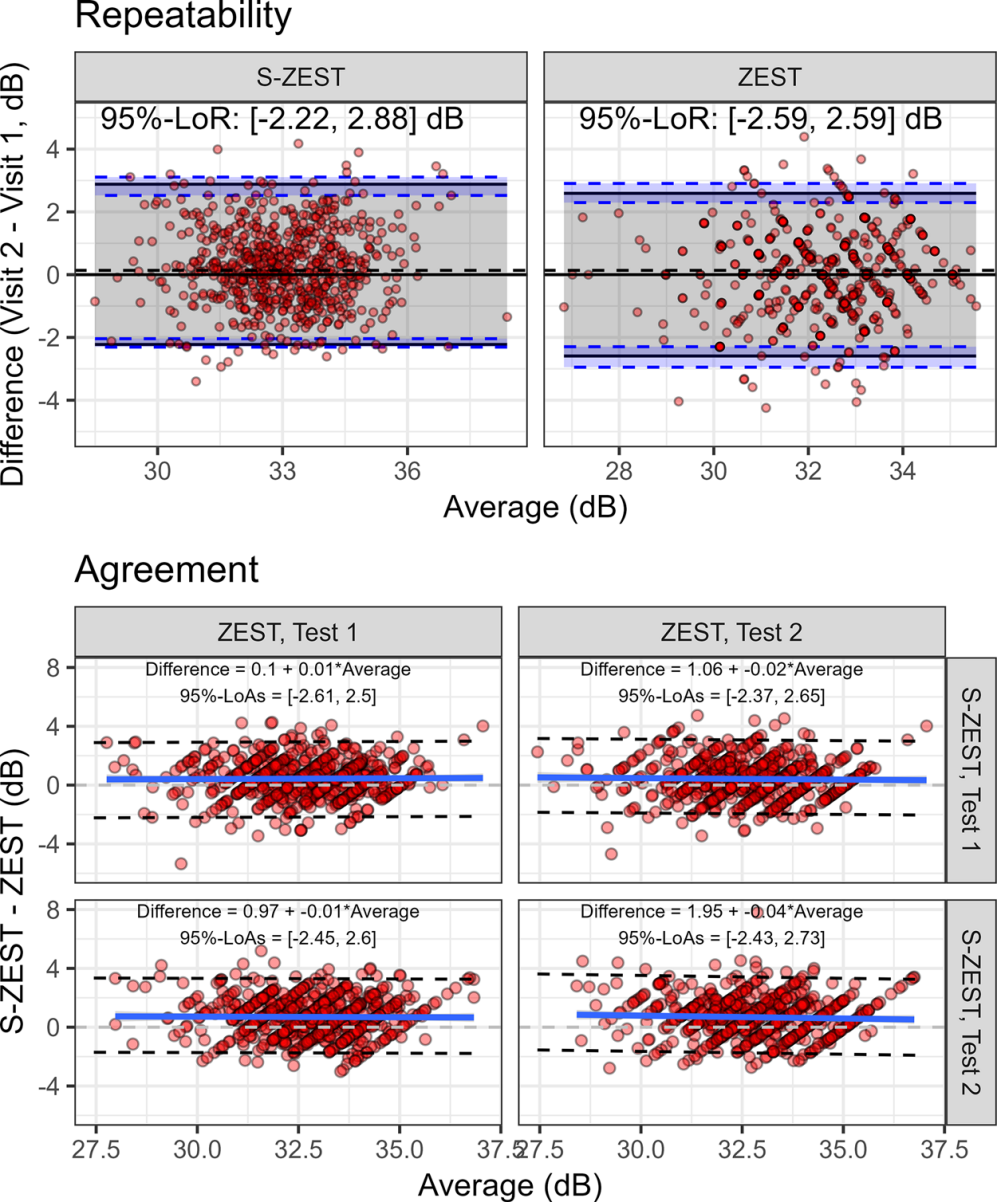
Bland-Altman plots for ZEST and S-ZEST. Top (repeatability): the solid lines represent the pointwise mean differences and 95% limits of repeatability; the dashed lines indicate the 95 confidence intervals of the LoRs (*blue shaded area*) and the line of no difference (*dashed horizontal line* on 0 dB). Bottom (agreement): pairwise test agreement, reporting the 95% limits of agreements and the bias equation.

The pointwise repeatability and agreement for the two strategies are reported in Figure 4. The 95% limits of repeatability for the S-ZEST were marginally narrower, but there was no statistically significant difference (*P* = 0.829). There was overall good agreement between the two strategies, with the 95% limits of agreement being very similar to the 95% limits of repeatability. There was no evidence of a proportional bias (all slopes for the bias equations were not significant). There was, however, a small but significant difference in pointwise sensitivity, which was higher with S-ZEST (estimate 0.56 dB; 95% CI, 0.35–0.77 dB; *P* < 0.001).

**Figure 5.**
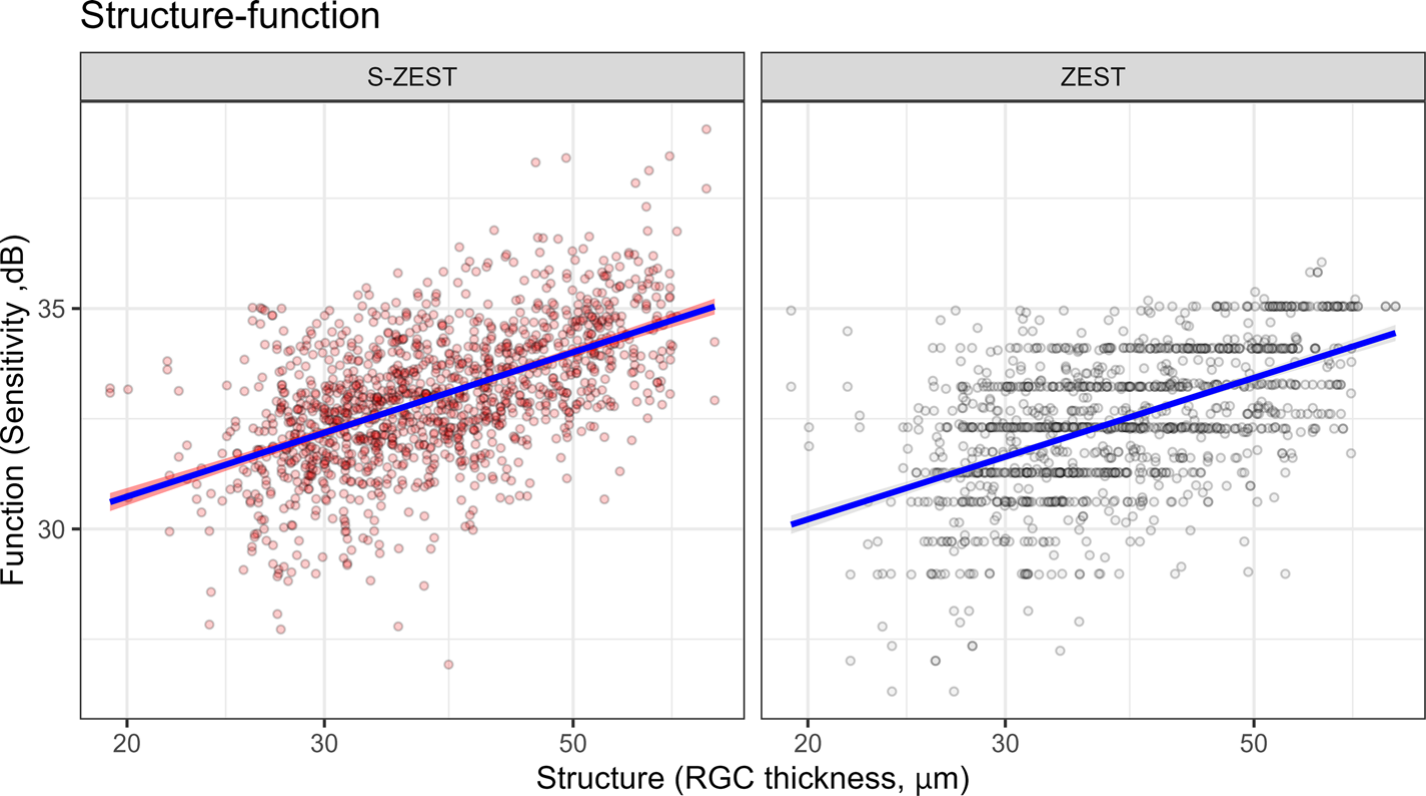
Structure-function relationship for ZEST and S-ZEST. Log10 spacing for the horizontal axis.

The structure–function correlation (Fig. 5) was significant (*P* < 0.001) and similar between the two strategies (no significant difference in slope, *P* = 0.862), with almost identical R2 values (0.24 and 0.23 for the S-ZEST and ZEST, respectively).

**Figure 6.**
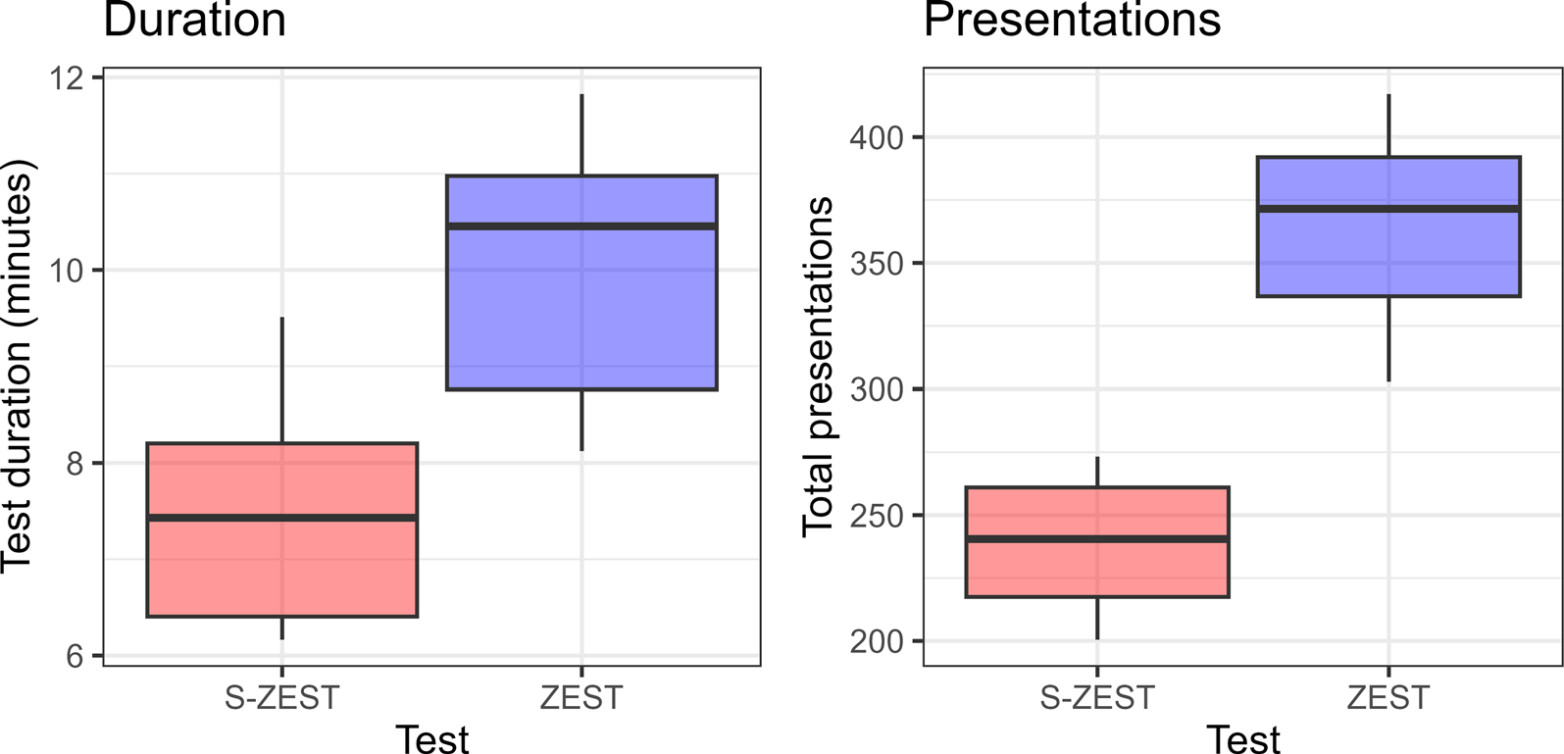
Box plots of test duration and number of presentations, reporting the median, interquartile range (box) and the 5% to 95% quantiles (whiskers).

There was a large, statistically significant difference in testing time between the two strategies (Fig. 6), with S-ZEST taking a mean (estimate, 7.5 minutes [95% CI, 6.6–8.3 minutes] to complete, compared with 10.1 minutes [95% CI, 9.5–10.8 minutes]) for ZEST (difference 2.7 minutes [95% CI, 1.3–3.7 minutes; *P* < 0.001). The number of presentations per test was also significantly reduced in S-ZEST (239 [95% CI, 219–259]) compared with ZEST (368 [95% CI, 345–391], difference: 129 [95% CI, 98–160]; *P* < 0.001).

### Usability

The average time taken to complete the main sequence was 52.9 ± 11.5 seconds (mean ± standard deviation), quantifying the additional time added to operate the web application. All participants were able to complete the sequence of tasks successfully, with the exception of one participant (clinician) who did not correctly select the S-ZEST for 1 out of 19 patients.

## Discussion

This study explores a practical framework that facilitates structurally enhanced perimetric examinations (S-ZEST) through an interactive application. Participants took less than 1 minute on average to complete the sequence of actions, which demonstrates the user-friendly nature of the application and supports the notion that this software could readily be integrated into a clinical or research setting. Furthermore, SD-OCT scans are already routinely used in a patient's glaucoma care.

In simulations, S-ZEST performed better in patients with glaucoma than in healthy subjects. This finding is expected, because the structure–function model, as implemented, was derived exclusively from patients with glaucoma owing to dataset availability. This strategy was preferable to providing an overly optimistic performance by fitting and testing the model on the same 10-2 cohort, used for simulations, which included healthy participants. Providing a more complete implementation of the structure–function strategy, which does include healthy subjects, is likely to eliminate the bias observed in simulations and will be a part of future development work.

It should be borne in mind that simulations can only offer a partial examination of the accuracy of structurally informed strategies, because the true structure–function relationship is not known. A further limitation is the small size of the dataset. In our simulations, we used the observed thresholds as a surrogate of the true threshold. However, observed thresholds are derived from a perimetric test and are, therefore, measured with noise. This is relatively unimportant when testing individual locations independently, but it becomes relevant when incorporating spatial models^22^ or structural data.^13^ Therefore, examining real participants needs to be the testing ground for these strategies, and our framework offers an important step to facilitate this.

The practical implementation tested ZEST without spatial enhancement and a S-ZEST with spatial enhancement. There were several rationales for testing the two extreme implementations of the strategy. First, these two implementations of the tests provided the closest accuracy in the simulated healthy observers. Second, spatially enhanced S-ZEST would still provide an improvement in speed compared with the spatially enhanced ZEST in patients with glaucoma, while retaining the same accuracy. Third, it would have been easier to optimize a strategy to perform extremely well in healthy subjects, exploiting the prior knowledge that they were healthy; however, this would have come at the expense of generalizability in patients with glaucoma. The simulations (Fig. 3) showed that the spatially enhanced ZEST introduced a positive bias in the estimates from patients with glaucoma, which was decreased by the spatially enhanced S-ZEST. Finally, we wanted to compare our fastest implementation of S-ZEST with an unbiased estimation of the threshold obtained with independent testing from a standard ZEST, as a means to assess the accuracy of our results. This should be kept in mind when considering the improvement in speed of the test. However, from our simulations, this comparison also seemed to provide the closest approximation of the difference in the number of presentations that would be observed in patients with glaucoma between spatially enhanced S-ZEST and ZEST. Therefore, we believe that these results have the greatest translational relevance for patients with glaucoma. Moreover, the spatial correlations in S-ZEST were customized using structural information and would not be replicated exactly in a nonstructurally informed strategy.

The results of the practical implementation show that, in visually healthy subjects, there was no significant difference in the repeatability of the measurements from the examinations, but the testing time was significantly reduced with S-ZEST (by approximately 26%). Testing a structurally informed strategy in healthy, young participants is not a validation of the strategy; however, the experiment does provide useful information about the performance. Structural data in healthy subjects contains information regarding the absence of disease and this information is exploited by S-ZEST to reduce the contribution of the abnormal component in the prior distribution, besides shifting the peak of the normal component. This aspect of S-ZEST can be tested in healthy subjects and is effective in decreasing testing time by restricting the effective prior range of the expected sensitivity.

By comparing the two strategies, ZEST and S-ZEST, we found a small but significant difference in the estimated sensitivity, which was on average 0.56 dB higher with S-ZEST. This result could be a consequence of the structure–function model. The model was developed from patients with glaucoma who were considerably older than the participants recruited for this study (see Table 1). This factor meant that the predictions of the model used to set the structural prior distributions were largely obtained by extrapolating outside the range of the data (both in terms of measured GCL thickness and age). This strategy could have caused an overestimation of the expected sensitivity values in the starting prior distributions. This issue can be overcome easily by implementing a structure–function model on a larger pool of patients and visually healthy people, and this objective will be explored in future work. It is interesting to note that this difference is opposite to what would be expected from the negative bias demonstrated by S-ZEST in the simulations. The estimates obtained with ZEST could have suffered from limitations in the normative model, derived from a much larger dataset, but based on a different testing pattern,^21^^,^^27^ which might not have been able to model fully the expected sensitivity, especially considering the young age of our participants. It should be noted that the age of the participants was, however, within the age range covered by the normative database in the validation study.^16^ In contrast, the S-ZEST was based on a continuous model of structural data and, as such, it showed greater flexibility in capturing the specific range of sensitivity of this specific cohort. Importantly, we showed that the average structure–function relationship was not significantly different between S-ZEST and ZEST. This analysis allowed us to verify, with an independent structural measurement, that S-ZEST did not introduce large distortions in the final results when compared with a ZEST agnostic to structural information.

Several attempts have been made to integrate structural information in VF testing procedures to improve perimetric examinations in glaucoma.^12^^–^^14^ These have achieved promising results, especially in decreasing testing time in simulation experiments. However, mainly because of technological limitations, practical implementations of these strategies have not been developed. This factor has prevented their testing on real patients and their deployment into clinical settings. Software solutions exist to extract imaging data from OCT instruments and to quantify structural parameters automatically. However, clinicians and technicians often require a user-friendly interface to perform data integration in clinical practice or when testing patients in a research environment. This innovation is the main advance of our work. We have developed a user-friendly web application based within the Shiny environment for R, which implements an automated data extraction of OCT data from raw files and performs real-time calculations of structural features to inform the perimetric strategy. The application is web based and can be used across different operating systems and computer platforms (https://github.com/giovmontesano/shinyCMP_OPI). Our usability assessment with 10 participants showed that, on average, the use of the application would increase the overall testing time by less than 1 minute. It should be noted that most of this time increase was due to processing, which could be substantially decreased with alternative coding approaches. This added duration does not account for the time taken to transfer the OCT data from the SD-OCT device to the computer connected to the CMP. However, this could easily be made virtually instantaneous by optimized networking of the different devices in clinical settings. In general, the application provided a user-friendly experience, demonstrated by the fact that virtually all 10 test users were able to complete the series of tasks, with a very low error rate (1/19 patients in 1/10 test users). Feedback from these test users highlighted areas of improvement. Participants commented that there were “too many steps” required and queried whether some of these could be automated. This issue can be addressed with future iterations. In this preliminary testing phase, the application was designed deliberately to obtain confirmation for steps crucial to the correct positioning of the testing locations, such as fovea detection and alignment of fundus images, which involved more actions from the user. General feedback was that the application required practice; however, one user commented it was “easy and straight-forward to use after 2–3 cases.”

A user-friendly interface is particularly helpful to implement structurally informed strategies to test the macular region with a fundus-tracked perimeter. Macular testing in glaucoma has risen to prominence in recent years with the widespread recognition that standard testing grids, such as the 24-2 grid, can overlook early macular defects because of their coarse spatial resolution.^9^^,^^28^ These defects can often be detected with a 10-2 grid. However, additional VF tests are time consuming and generate an additional burden for both patients and clinicians. Combined testing grids, which add macular testing locations to the standard 24-2 grid, have been proposed as a practical compromise.^27^^,^^29^ Another possibility would be to guide the acquisition of macular tests with structural information; OCT scans could be used to decide the position of additional testing locations^28^ or to improve the macular test with customized prior information.^12^ In this current work, we implemented the latter strategy. The previous literature^30^^–^^33^ has outlined the benefit of customized location selection, in particular increasing spatial sampling resolution in regions of deficit. Using OCT to inform location selection in such situations would be another beneficial application of this framework.

One advantage of using a fundus-tracking perimeter, such as the CMP, is that the OCT scans can be aligned precisely with the fundus image used to track the eye movements during the test. On the one hand, this allows for a more precise structure-function mapping. On the other hand, it could help with standardization by setting the position of the testing grid based on consistent anatomical landmarks, such as the fovea or the fovea–disc axis. This approach was chosen for our implementation. Although this approach is unlikely to provide great benefit in healthy observers, it could become helpful in patients with unstable or eccentric fixation,^34^ or with unusual anatomy.

The greatest limitation of our work was the structure–function model itself. Although this metric was not the main focus of this work, better structure–function predictions could certainly be achieved with more advanced methods, such as artificial intelligence.^35^^–^^38^ The improved accuracy of the structure–function model is expected to provide a larger improvement in both speed and accuracy of the test.^13^ Our group has shown, with simulations, that artificial intelligence predictions can be incorporated easily into perimetric tests using the same framework adopted for this work, with improvements in both speed and accuracy.^39^

Our strategy only relied on local measurements of GCL thickness, corrected for GC displacement. Artificial intelligence could also be used to improve layer segmentation or to detect features of diseases other than glaucoma at the macula. This factor could help to determine better and more customized prior distributions that could account for multiple diseases and reduce the risk of bias in structurally informed perimetric tests. A broader discussion should focus on whether improving structure–function correlation deprives clinicians of useful information obtained from structure-function disagreement. For example, clinicians might use structural and functional tests as independent sources of information to detect progression. However, if the two correlate more strongly because of specific implementations of the perimetric tests, structural noise is also likely to influence the functional assessment. This factor should be taken into account when introducing these strategies into the clinic. An alternative, as discussed elsewhere in this article, could be to use structural information to add bespoke testing locations, which can then be tested with structurally agnostic strategies. This would allow clinicians to confirm suspected structural defects with a perimetric assessment independently. Our framework could be modified easily to account for this possibility, taking full advantage of the spatial precision offered by fundus tracking technology.

In conclusion, our team has developed a practical framework to facilitate structurally enhanced perimetric examinations, which has been shown to improve test duration without compromising on accuracy, using a user-friendly and quick web-based application. Future developments will focus on extending this approach outside the macular region (for example by using the retinal nerve fiber layer profile or wide field OCT scans), testing the framework on a large pool of patients with glaucoma, and improving the model used for structure–function predictions.

## Supplementary Material

Supplement 1
