# Supplementary material for: A Practical Framework for the Integration of Structural Data Into Perimetric Examinations
Source: Transl Vis Sci Technol. 2024 Jun 25;13(6):19. doi: 10.1167/tvst.13.6.19 (PMC11205229; doi:10.1167/tvst.13.6.19)
Supplement: Supplement 1 [file tvst-13-6-19_s001.pdf]

Supplementary Material

Standard prior distributions

The subject's age was taken into account when determining the starting prior probability density function (PDF). A normative regression line of sensitivity vs age was available for each location in the grid used to collect the normative dataset (24-2 with 12 additional macular locations). The normative values for the 10-2 locations were then calculated by linearly interpolating those from the available locations. **Figure 1** is an example of the different prior PDFs in a patient with glaucoma.

Structure-function model

The structure-function model was based on a dataset of 19 patients with glaucoma who had performed a test-retest session with a CMP perimeter using a 24-2+ grid [22], which tests all the 52 locations normally analysed in a 24-2 grid and 12 additional macular locations. These tests were performed for a separate study to develop a progression algorithm for the CMP. The original dataset contained 90 eyes from 90 patients, but macular scans performed with the Spectralis SD-OCT were available to us for only 19/31 patients recruited at IRCCS Fondazione "G. B. Bietti" (Rome, Italy). These were used to fit the model. This sample had the following characteristics, reported as average (standard deviation): age: 68 (6) years; 24-2 MD: -7.5 (5) dB; 24-2 PSD: 9.2 (3.4) dB.

The macular scan was composed of 61 B-scans (9 averaged images), spanned 30 x 25 degrees and was aligned with the fovea-disc axis and centred on the fovea. The local average RGC thickness was calculated from the exported `.vol` files for each one of the 24 macular locations in the 24-2+ grid, using the displacement method published by Montesano et al. [26], after aligning the fundus images from the CMP with the SLO from the Spectralis. The structure function model was a simple linear model with pointwise sensitivity as a response variable, and the local thickness and subject's age as covariate. The model was fitted with the *crch* package for R [41], which is able to account for censored data (i.e. sensitivity measurements below the 0 dB floor) and to model heteroskedasticity using a log-link function for the variance with the same predictors as the mean. The variability of the response variable (sensitivity) was modelled with a logistic distribution because this gave the best fit for the data. Other models, such as a linear-linear model [42] and one including eccentricity and various interactions, were also tested but did not provide any additional improvement to the prediction. As expected, the prediction was extremely poor below 20 dB. To overcome this issue, we fitted a logistic regression to model the proportion of values < 15 dB as a function of the deviation for the normative age-corrected value for the same location (i.e. the total deviation of the prediction, TDP). The normative reference values were obtained from the data in Montesano et al. [17]. As expected, this proportion increased as a function of the TDP. The predictions from this logistic regression were used to generate varying weights for the 'abnormal' peak in the prior distributions for S-ZEST (see next paragraph). Correlations among data from the same eye and locations were ignored because we were only interested in population-level predictions and because the design was perfectly balanced (all eyes had 6 test values for 24 macular locations).

Structurally informed prior distributions

The prior distributions for S-ZEST were built in a similar way to the standard ZEST. The main difference was that the peak of the 'normal' component was centred on the prediction from the structure-function model rather than on the normative reference value. The proportions used to combine the 'normal' and 'abnormal' components were also modified based on the TDP. The weight for the 'normal' component was calculated as the inverse of the prediction from a logistic regression modelling the proportion of values < 15 dB (obtained on the training data, see previous paragraph), meaning that it would increase for structural predictions closer to the expected normative reference value (i.e. TDP closer to 0 dB). This weight was not allowed to be lower than 4, the fixed value set for the standard ZEST. This meant that for lower structure-function predictions, the mixing proportions were identical to ZEST (but with a different location for the peak). For locations with predictions closer to the normative value, the proportion would instead favour the 'normal' component in the mixing.

Simulations

The numerical values for the simulation results illustrated **Figure 3** are outlined in **Supplementary Material Table 1**.

			**Reliable**		**High false positives (20%)**		**High false negatives (20%)**	
			**MAE (dB)**	**Presentation**	**MAE (dB)**	**Presentation**	**MAE (dB)**	**Presentation**
**Healthy**	**ZEST**	**w/o SE**	1.35 [1.04, 1.98]	292 [268, 350]	1.41 [1.07, 2.3]	298 [273, 337]	2.53 [1.79, 3.49]	321 [292, 383]
		**w/ SE**	1.2 [0.92, 1.75]	249 [221, 309]	1.31 [1, 2.15]	249 [221, 297]	1.94 [1.35, 2.83]	281 [247, 345]
	**S-ZEST**	**w/o SE**	1.72 [1.31, 2.33]	295 [262, 358]	1.78 [1.36, 2.35]	301 [266, 377]	2.83 [2.00, 4.27]	300 [272, 340]
		**w/ SE**	1.43 [1.11, 1.84]	264 [227, 319]	1.5 [1.15, 2.03]	271 [232, 335]	2.34 [1.64, 3.23]	277 [240, 327]
**Glaucoma**	**ZEST**	**w/o SE**	1.99 [1.45, 2.85]	379 [326, 448]	3.87 [2.14, 6.66]	388 [321, 463]	2.51 [1.42, 3.64]	388 [346, 446]
		**w/ SE**	1.67 [0.99, 2.52]	333 [279, 424]	2.79 [1.77, 4.37]	360 [291, 439]	2.2 [0.94, 3.43]	342 [268, 419]
	**S-ZEST**	**w/o SE**	2.09 [1.50, 3.00]	336 [285, 403]	3.6 [2.04, 6.46]	344 [288, 417]	2.69 [1.53, 3.85]	338 [293, 391]
		**w/ SE**	1.7 [0.99, 2.70]	296 [242, 382]	2.47 [1.51, 3.95]	325 [262, 405]	2.35 [0.96, 3.67]	294 [227, 371]

Supplementary Material Table 1: Performance of ZEST and S-ZEST in simulations (500 per test). The results are reported as Median [2.5%, 97.5%] quantiles. SE = spatial enhancement. MAE = Mean Absolute Error per test, average of the pointwise AE.

Difference in sensitivities between strategies

The differences between the mean of the starting prior distribution and the final sensitivity are plotted in **Supplementary Material Figure 1**. This combines the effect of location of the 'normal' peak and the scaling of the 'abnormal' distribution. The prediction is the normative value for ZEST and the structural prediction for S-ZEST.

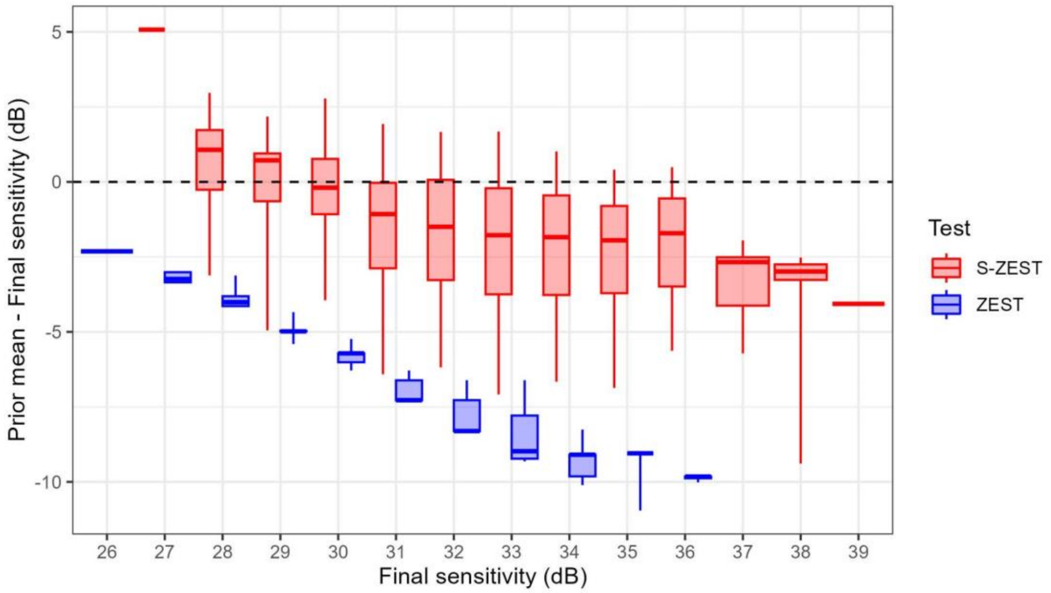

Supplementary Material Figure 1: The difference between the mean of the starting prior distribution and the final sensitivity.
